# Profiles of Teacher Professional Identity Among Student Teachers and Its Association With Mental Health

**DOI:** 10.3389/fpubh.2022.735811

**Published:** 2022-04-06

**Authors:** Shunying Zhao, Yang Dong, Jian Luo

**Affiliations:** ^1^School of Education Science, Academic Affairs Office, Jiaying University, Meizhou, China; ^2^Department of English, Hainan University, Haikou, China

**Keywords:** teacher professional identity, student teachers, latent profile analysis, psychological wellbeing, anxiety

## Abstract

In recent years, studies exploring the link between teacher professional identity and mental health are increasing. However, such research using latent profile analysis is still scarce. The aim of this study was to examine the heterogeneity of a sample of 923 student teachers' professional identity and its association with mental health (i.e., psychological wellbeing and anxiety). By using latent profile analysis, four different profiles were identified: (1) low professional identity (LPI, 5.1%), (2) moderate professional identity (MPI, 42.7%), (3) high occupational values (HOV, 12%), and (4) high professional identity (HPI, 40.2%). The results demonstrated that psychological wellbeing and anxiety were significantly different across the four profiles. In addition, the anxiety of HOV group was not significantly with LPI and MPI groups. This study indicated that student teachers in different groups may have different mental health status.

## Introduction

Student teachers are prospective teachers in the Chinese educational context. Most of student teachers must spend 3 or 4 years to finish their teacher education, which includes subject major courses, teaching knowledge, and skill courses (1). The education and training student teachers received during university revolves around how to become a qualified teacher. However, most student teachers in China are uncertain about whether they will enter the teaching profession after graduating from university. For instance, Wang and Zhang (2) found that only 47% student teachers chose the teaching profession as their first career choice. One reason may be that student teachers have not yet formed teacher professional identity well-before graduating from college.

Social identity theory (3), a theoretical basis of identity research, indicated that social identity formation in an intergroup context contains four processes: (a) social categorization, (b) the formation of an awareness of social identity, (c) social comparison, and (d) a search for psychological distinctiveness (4). Professional identity, a kind of social identity, refers to having a cogent view of one's stable pattern of career goals, interests, strengths, and potentialities to make positive career-related decisions with greater self-assurance (5, 6). Professional identity is an individual's attitude and sense of devotion to a profession, which is reflected in individual's desire to continue working in the profession and their degree of liking (7). The professional identity of student teachers is the determining factor of motivation toward, satisfaction with and commitment to teaching profession, which is also an important factor for primary and secondary schools to consider when employing teachers (8). Even though the research on developmental course of a student teachers' professional identity is somewhat sparse, research applied social identity theory to teacher professional identity are very rich. For example, recent quantitative studies have explored the first-year college students' (9), and music teachers' (10) professional identity development according to social identity theory. A qualitative study reviewed middle and high school science teachers' identity at the perspective of social identity theory (11). To fill this gap, this study tried to explore student teachers' professional identity by social identity theory.

Unlike Western countries, teachers' social status in China is relatively higher. According to the 2018 Global Teacher Status Index, Chinese teachers' social status ranked seven among the 35 surveyed countries, while the US, France, and UK ranked 18, 14, and 12, respectively (12). In Chinese history, respecting teachers and valuing education is a traditional virtue of Chinese people, which means that the teaching profession is regarded as a highly respected and high-status profession (13). With that in mind, it is rather puzzling that many Chinese student teachers have a low professional identity (14–16). Specifically, 57% of them are not sure whether teachers have a promising career and whether they are suitable to be teachers; 74% are not sure whether they will always be teachers after they graduate; 61% still don't understand the social significance of the teaching profession thoroughly; 53% are not sure whether they are able to be a good teacher (17). Besides, previous studies point out that individuals' low level of professional identity may have links with their mental health status (18). Therefore, it is necessary to identify subgroups of student teachers according to their teacher professional identity, and explore how to promote student teachers' professional identity. By investigating these issues, target training of teacher professional identity among teacher candidates may be possible.

## Person-Centered Approach in Teachers' Professional Identity Research

In general, previous studies on teacher professional identity tend to adopt a variable-centered approach (13, 19), which tends to focus individuals' whole level of each dimension and its' relationships with antecedents or outcomes. However, this approach ignores the heterogeneity within each level (20) and hence is unlikely to reflect reality (21). It also assumes that all individuals belong to either a single group or multiple subgroups with known subgroup memberships (e.g., gender), which can't address the concern of whether several subgroups represent different classes (19). Unlike variable-centered approach, a person-centered approach can dress this problem well. According to individuals' response patterns on a set of variables, a person-centered approach can identify emergent subgroups that show the same pattern (i.e., profile) of behavior (22). For this reason, this study adopted person-centered approach (i.e., latent profile analysis) to understand the heterogeneity in student teachers' professional identities.

To date, only one study (23) used traditional person-centered approach (i.e., hierarchical cluster analysis), which found three subgroups of teachers with different identity—characterized as (a) teachers with positive professional identity, (b) teachers with negative professional identity, and (c) uncommitted teachers. Unlike hierarchical cluster analysis, latent profile analysis (LPA) is a new person-centered approach that sorts individuals into groups of subjects who are similar to each other and different from the other groups according to their value indicators (24). Although LPA is similar to hierarchical cluster analysis in terms of its goal, many scholars have pointed out that LPA is more practical and has more power for detecting optimal number of profiles (25). Besides, LPA is more objective and flexible than cluster analysis due to the probabilistic classification (26). Specifically, it classifies heterogeneous groups, estimates all possibilities of individuals belonging to a certain type, performs model fitting with various fit indicators, determines the number of categories based on those indicators, and examines complex relationships among variables (26–28). Student teachers are at the formation stage of teacher professional identity, which make their characteristic psychological states and behavioral patterns may different from in-service teachers. Therefore, we cannot directly apply Karaolis and Philippou's (23) research results to student teachers in college. To fill this gap, we investigated sub-populations of student teachers using LPA. We expected to provide a new perspective on the professional identification theories of student teachers and some suggestions for training student teachers' professional identity.

## Student Teachers' Professional Identity and Mental Health

In recent years, China has continuously reformed in primary and secondary schools. This situation forwards new demands on the ability of teachers, which in turn increased the requirements of student teachers' abilities and accomplishments. Therefore, student teachers experience more anxiety and less psychological wellbeing during university, which lead to serious mental health problems among them (29). Besides, Chinese parents and teachers usually attach great importance to students' academic performance. This situation creates extra psychological burdens on student teachers who don't like their major, which may not only waste social education resources but also threaten student teachers' mental health. To improve student teachers' professional identity effectively, it is necessary to identify teacher professional identity profiles and explore its associations with mental health indicators.

Previous research points out that individuals' mental health status should be assessed by two independent but correlated concepts (i.e., positive mental health and mental disorders) (30, 31). In this view, positive mental health (i.e., mental wellbeing) and mental disorder (i.e., mental health problems, psychopathology or negative wellbeing) are required for complete mental health assessments and should be integrated in one research (31). Besides, there are many studies used psychological wellbeing and anxiety to measure individuals' mental health (32–34). For this reason, this study chose psychological wellbeing (as indicator of positive mental health) and anxiety (as indicator of mental disorder) to measure mental health.

Psychological wellbeing refers to “striving for perfection”—the realization of one's true potential (35)—which suggests that the ability to search for valuable professional opportunities growth likely contributes to profession exploration (36). Studies indicates that individuals' professional identity has a positive relationship with their psychological wellbeing (37–40). Zhou and Xu (41) indicate that university students with higher professional identity may have higher psychological wellbeing. Professional identity may serve as an internal compass for guiding and developing a career in an unpredictable job market and in the absence of proper guidelines for career development (42). As a predominant source of meaning in life (43), professional identity can help individuals produce psychological wellbeing. Therefore, it can be reasonably to hypothesize that a profile with a relatively high professional identity would have a relatively high psychological wellbeing.

In contrast, previous research suggests that student teachers with low professional identity are more likely to experience anxiety during university (44, 45). Whether in China or other countries, almost all student teachers must gain experience through several months of student teaching in primary or secondary schools as part of their college education. When they participate in teaching practice in elementary or middle schools, they often experience anxiety (46). Therefore, we hypothesized that profiles with stronger professional identity would correspond to a weaker state anxiety. Besides, previous research points that gender (47–49) and regional differences (14, 50) may play important roles in the formation of teacher professional identity. Exploring these two factors among student teachers may also help to reveal the formation mechanism of their professional identity.

## The Current Study

The aims of this study are 2-fold. First, this study tried to use LPA to identify how many profiles exist among the current sample. Based on the four dimensions of teacher professional identity's independence (24, 51, 52), this study expected to find at least two profiles: (a) low levels in all four dimensions of professional identity and (b) high levels in all four dimensions of professional identity. This study also tried to summarize the response patterns and mindset of each subgroup separately. To test the validity of the profile results, this study also compared the differences in mental health indicators (i.e., psychological wellbeing and state anxiety) across different profiles. Second, this study discussed the relationship between student teacher professional identity and mental health indicators (i.e., psychological wellbeing and anxiety).

The current study answered the following questions:

Q1. How many profiles on teacher professional identity among the current research sample? What are the response patterns and mindset of each subgroup separately?
Q2. Are there any differences in mental health indicators across different profiles?

This study tested the following two specific hypotheses:

Hypothesis 1. There are two teacher professional identity profiles among this sample at least: (a) low levels in all four dimensions of professional identity, and (b) high levels in all four dimensions of professional identity.
Hypothesis 2. Profile with a relatively high professional identity would have a relatively high psychological wellbeing but have a relatively low anxiety.

## Methods

### Participants

The survey for this study was conducted from March 18 to 21, 2020. Participants were a sample of 970 student teachers from two local universities with similar training and teaching plans in Guangdong Province, China. To abide by the policy of the university ethics committee of the first author, we distributed the questionnaire to potential participants electronically via SurveyStar (Changsha Ranxing Science and Technology, Shanghai, China), and no face-to-face contact was made. After excluding invalid data (i.e., regular response), 923 participants (*M*_age_ = 20.84, *SD*_age_ = 1.62, range_age_ = 18–26; 72.4% female; 24.1% 1st year, 39.7% 2nd year, 23% 3rd year, and 13.3% 4th year; 38.6% urban areas, and 61.4% rural areas) were included in the final analyses. Every participant volunteered to participate in this study and received no compensation.

### Measures

#### Professional Identity

Student teachers' professional identity was measured by teachers' professional identity scale (53) with 18-items (e.g., “I am proud of being a teacher”) consisting of four subscales: (1) six items of role value, (2) five items of professional behavior inclination, (3) four items of occupational value, and (4) three items of occupational belonging sense. Previous studies indicated that the original scale has a good reliability and validity (53–55). Each item is scored from 1 (very strongly disagree) to 5 (very strongly agree). To adapt this scale to student teachers, this investigation revised some items (e.g., “I am proud to be a teacher in the future”).

#### Psychological Wellbeing

Psychological wellbeing was measured by the flourishing scale, which was originally developed by Diener et al. (56). The original scale consists of eight items and describes essential aspects of human functioning regarding individual fulfillment or self-actualization. The questionnaire was scored on a 7-point Likert scale, ranging from 1 (very strongly disagree) to 7 (very strongly agree), with a higher score suggesting more psychological wellbeing. The Cronbach's α of the original scale was 0.85 (57). The Cronbach's α of the Chinese version was 0.95 (58).

#### State Anxiety

This study used Zung's (59) self-rating anxiety scale to assess student teachers' anxiety, which has 20 items (e.g., “I feel more nervous and anxious than usual”). The scale is a four-point scale, ranging from 1 (a little of the time) to 4 (most of the time), to evaluate how frequently the symptoms described by the items occur. The Chinese version of the SAS was widely used and has good reliability and validity (60, 61).

### Data Analysis

First, data screening was used to exclude invalid data (e.g., participants completed the survey within 90 s). Second, SPSS 20.0 was used to calculated descriptive statistics. Tests of normality revealed that the study variables showed no significant deviation from normality (i.e., Skewness < |2.0| and Kurtosis < |7.0|, see Table 1) (62). Third, we used Mplus version 7.0 for LPA to identify the number of latent profiles of student teachers' professional identity that best fit the data. We tested models starting with two profiles and gradually increased the number until the model fit no longer showed significant improvement. We used log-likelihood (i.e., LL), Bayesian Information Criterion (i.e., BIC), Akaike Information Criterion (i.e., AIC), and the sample-size adjusted BIC (i.e., SABIC) to evaluate the best fitting LPA model (63). Entropy was examined in the present study to evaluate the accuracy of model classification. A higher value of entropy (i.e., above 0.8 and closer to 1) indicates better latent profile separation (64). Finally, one-way ANOVA conducted by SPSS 20.0 was used to examine the differences in the mean levels of psychological wellbeing and anxiety in different latent profiles.

**Table 1:** Means, standard deviations, and correlations of the main study variables.

	***M***	***SD***	**1**	**2**	**3**	**4**	**5**	**6**	**7**
1. RV	4.06	0.68	1						
2. PBI	4.23	0.58	0.71***	1					
3. OV	4.46	0.55	0.58***	0.70***	1				
4. SOB	4.00	0.72	0.50***	0.49***	0.43***	1			
5. TPI	4.18	0.52	0.85***	0.87***	0.80***	0.76***	1		
6. PWB	5.41	0.90	0.59***	0.59***	0.43***	0.39***	0.61***	1	
7. Anxiety	1.85	0.38	−0.13***	−0.16***	−0.09*	−0.01	−0.12**	−0.24***	1
8. Skewness	–	–	−0.70	−0.40	−0.54	−0.76	−0.38	−0.14	0.90
9. Kurtosis	–	–	0.68	−0.01	−0.49	1.26	−0.10	−0.18	2.39

M, mean; SD, standard deviation; N = 923; RV, Role values; PBI, Professional behavior inclination; OV, Occupational values; SOB, Sense of occupational belonging; TPI, Teacher professional identity; PWB, Psychological wellbeing. ***p < 0.001; **p < 0.01; *p < 0.05.

## Results

### Preliminary Analyses

Table 1 shows the means, *SDs*, and Pearson correlations for the main research variables. As the results showed, teacher professional identity and its four dimensions (i.e., role values, professional behavior inclination, occupational values, and sense of occupational belonging) were positively correlated with psychological wellbeing and negatively correlated with anxiety. There was no significant correlation between sense of occupational belonging and anxiety. In this study, the Cronbach' s α of professional identity was 0.94. For each sub-scale, the Cronbach's α of role value sub-scale was 0.89, professional behavior inclination sub-scale was 0.88, occupational value sub-scale was 0.91, and sense of occupational belonging was 0.81. Furthermore, CFA showed acceptable fit for this four-factor scale, TLI = 0.93, CFI = 0.94, RMSEA = 0.075, SRMR = 0.051. Besides, the Cronbach' s α of psychological wellbeing was 0.94, and the state anxiety was 0.83.

### LPA Profiles

Table 2 presents the results of LPA for teacher professional identity's four dimensions levels. With the addition of latent profiles, the information criteria BIC, AIC, and SABIC consequently decreased. The AIC, BIC, and SABIC decreased sharply from the three-profile solution to four-profile solution and showed no substantial drops in the following models. Therefore, the four-class solution was supported by all three information criteria values. Furthermore, the LMR and BLRT statistics were also significant in four-profile structure. According to the graphic presentation of the information criteria, this study should accept a four-profile solution.

**Table 2:** The model selection criteria of LPA.

**Model**	***LL***	**^**#**^ *P***	**AIC**	**BIC**	**SABIC**	**LMR (P)**	**BLRT (P)**	**Entropy**	**Class size: *n***
2-Profile	−2859.42	13	5744.85	5807.61	5766.32	0.000	0.000	0.90	457/466
3-Profile	−2555.91	18	5147.82	5234.72	5177.55	0.000	0.000	0.98	48/407/468
**4-Profile**	–**2410.03**	**23**	**4866.05**	**4977.09**	**4904.04**	**0.001**	**0.000**	**0.93**	**47/394/111/371**
5-Profile	−2314	28	4683.99	4819.16	4730.24	0.103	0.000	0.94	45/375/118/23/362
6-Profile	−2203.03	33	4472.05	4631.36	4526.65	0.258	0.000	0.94	19/49/81/97/349/328

*N = 923. Bold values represent a best-fitting model*.

**Figure 1:**
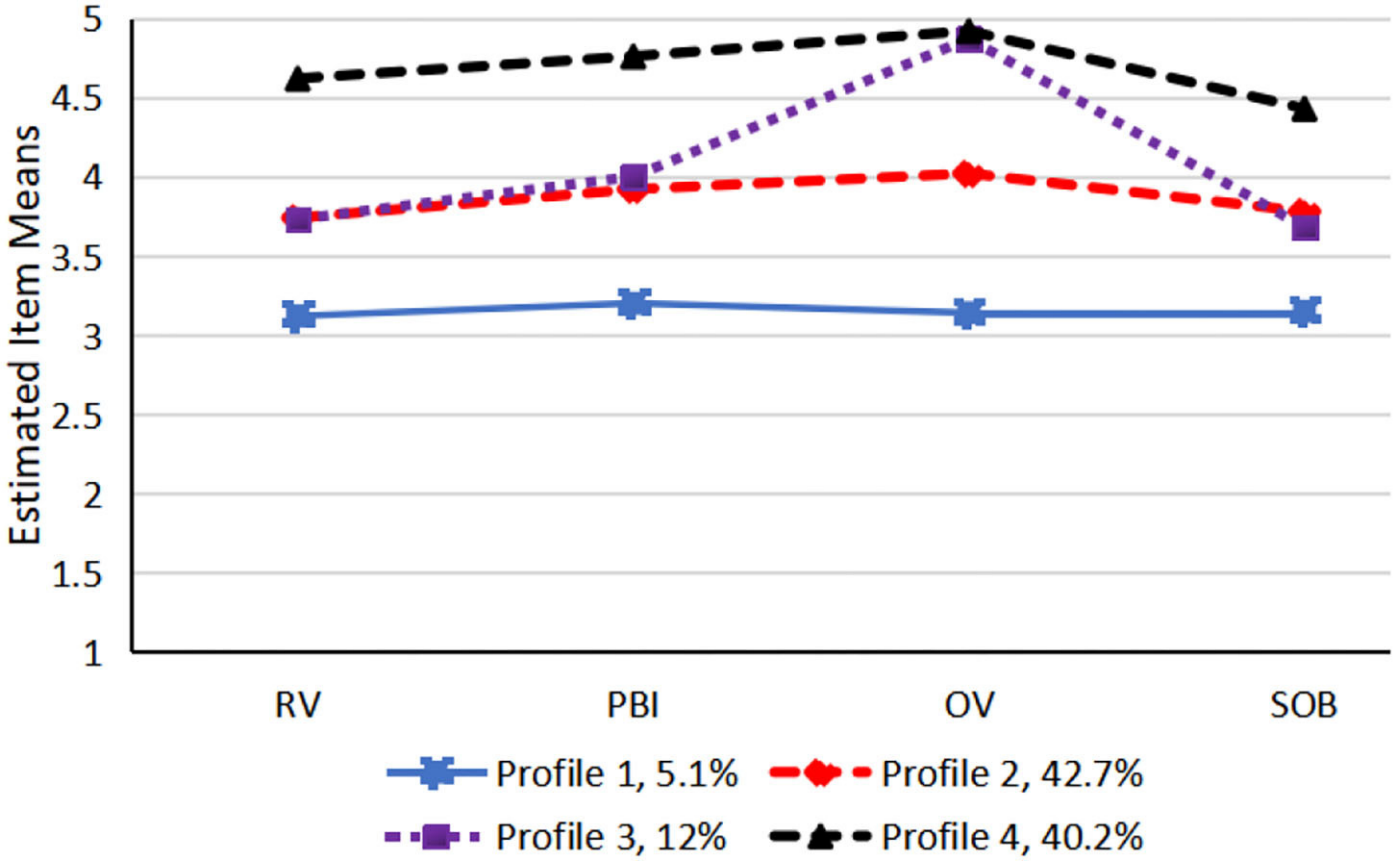
Four-profile solution of teachers' professional identity. RV, role values; PBI, professional behavior inclination; OV, occupational values; SOB, sense of occupational belonging.

Figure 1 plots the estimated mean of each teacher professional identity's dimension for the four latent profiles. Table 3 describes the detailed characteristics of each group. Together, the means of these dimensions provide a description of what characterizes each teacher professional identity profiles of participants. The profile 1 (5.1%) was characterized by tending to choose option 3 (i.e., I am not sure) for each item. Therefore, individuals in this profile often have a vague identification. Thus, we named this profile the low professional identity group (LPI). Student teachers in LPI may not agree with social significance of teacher profession. They may not regard themselves as teacher candidates. They may unlikely to experience the sense of sharing camaraderie with teacher groups and are reluctant to attend teacher education projects and courses. Profile 2 (42.7%) was labeled the moderate professional identity group (MPI) due to the moderate scores of each dimension. In profile 2, student teachers have a moderate degree with teacher profession identity. Since participants in Profile 3 (12%) have a rather high score on occupational value but moderate scores on other three dimensions, profile 3 (12%) were labeled the high occupational values group (HOV). Student teachers in HOV may evaluate teachers' work, agree with the opinion like that “I think that teachers' work is very important for students' development and accomplishment”. Finally, profile 4 (40.2%) was named the high professional identity group (HPI). Individuals in HPI have the highest scores on each four dimensions. In HPI group, student teachers may evaluate social significance of teacher profession highly. They may will to regard themselves as teacher candidates. They may tend to have positive emotional experience to be a teacher in the future. To be competent for teaching work in the future, they may participate in teacher training plan without much encouragement.

**Table 3:** Characteristics of the four teacher professional identity groups.

	**Participants *n* (%)**	**Characteristics**
LPI	47 (5.1%)	Low scores across all teacher professional identity indicators.
MPI	394 (42.7%)	Moderate levels in all indicators of teacher professional identity.
HOV	111 (12%)	High occupational values, but moderate levels in all other indicators of professional identity.
HPI	371 (40.2%)	High levels in all indicators of teacher professional identity.

*RV, Role values; PBI, Professional behavior inclination; OV, Occupational values; SOB, Sense of occupational belonging; TPI, Teacher professional identity; PWB, Psychological wellbeing; LPI, low professional identity group; MPI, moderate professional identity group; HOV, high occupational values group; HPI, high professional identity*.

### Relationship Between LPA Membership and Outcome Variables

One-way ANOVA was used to examine the validity of the four latent profiles again and compare the differences in psychological wellbeing and anxiety (see Table 4).

**Table 4:** Differences in means of teacher professional identity, psychological wellbeing and anxiety across latent profiles.

	***F***	**LPI (1)**		**MPI (2)**		**HOV (3)**		**HPI (4)**		***Post-hoc***
		**M**	**SD**	**M**	**SD**	**M**	**SD**	**M**	**SD**	
Role values	318.16***	3.11	0.50	3.74	0.53	3.68	0.60	4.62	.35	1 <2***; 1 <3***; 1 <4***; 2 = 3; 2 <4***; 3 <4***
PBI	576.06***	3.19	0.41	3.92	0.35	3.96	0.43	4.76	0.29	1 <2***; 1 <3***; 1 <4***; 2 = 3; 2 <4***; 3 <4***
OV	3389.48***	3.13	0.23	4.00	0.14	4.85	0.21	4.92	0.16	1 <2***; 1 <3***; 1 <4***; 2 <3***; 2 <4***; 3 <4***
SOB	116.00***	3.14	0.55	3.79	0.56	3.67	0.80	4.42	0.62	1 <2***; 1 <3***; 1 <4***; 2 = 3; 2 <4***; 3 <4***
TPI	919.76***	3.14	0.31	3.86	0.27	4.04	0.29	4.68	0.22	1 <2***; 1 <3***; 1 <4***; 2 <3***; 2 <4***; 3 <4***
PWB	137.99***	4.39	0.66	5.13	0.68	4.90	0.84	5.98	0.80	1 <2***; 1 <3***; 1 <4***; 2 <3**; 2 <4***; 3 <4***
Anxiety	7.12***	2.02	0.46	1.86	0.39	1.91	0.34	1.79	0.36	1 > 2**; 1 = 3; 1 > 4**; 2 = 3; 2 > 4*; 3 > 4**

M, mean; SD, standard deviation; N = 923; LPI, low professional identity group; MPI, moderate professional identity; HOV, high occupational values group; HPI, high professional identity; *p < 0.05. **p < 0.01. ***p < 0.001.

The mean of teacher professional identity varied across the four profiles [*F*_(3, 923)_ = 919.76, *p* < 0.001]. Role values' mean varied across the four profiles [*F*_(3, 923)_ = 318.16, *p* < 0.001]. The mean levels of professional behavior inclination varied across the four profiles [*F*_(3, 923)_ = 576.06, *p* < 0.001]. HPI student teachers' professional behavior inclination was significantly higher than other three profiles. Student teachers' occupational values varied across the four profiles [*F*_(3, 923)_ = 3,389.48, *p* < 0.001]. Finally, the mean difference in occupational belonging across the four profiles was also significant [*F*_(3, 923)_ =116, *p* < 0.001].

Finally, the mean difference in psychological wellbeing across the three profiles was significant [*F*_(3, 923)_ = 137.99, *p* < 0.001]. Specifically, the psychological wellbeing of HPI profile was significantly higher than other three profiles. The HOV profile was significantly higher than MPI and LPI profile. The MPI profile was significantly higher than LPI profile. Besides, the mean difference in anxiety was also significant among the four *profiles* [*F*_(3, 809)_ = 7.12, *p* < 0.001]. Table 4 showed that the anxiety of LPI profile was significantly higher than MPI profile and HPI profile. There was a marginal significance between LPI profile and HOV profile (*p* = 0.071). The MPI profile and HOV profile were significantly higher than HPI profile. Unexpectedly, there was no significance between the MPI profile and the HOV profile.

### Supplementary Analyses

Although analyzing demographic variables (i.e., gender and regional differences) is not the purpose of this study, testing these differences may be helpful. In the supplementary analyses, we tested gender and regional differences using independent sample *t*-test by SPSS 20.0 (see Tables 5, 6).

**Table 5:** Gender differences in means of teacher professional identity, psychological wellbeing and anxiety.

	***N***	**TPI**		***t***	**PWB**		***t***	**Anxiety**		***t***
		**M**	**SD**		**M**	**SD**		**M**	**SD**	
Male	255	4.08	0.54	−3.61***	5.51	0.85	2.14*	1.81	0.47	−1.81
Female	668	4.22	0.51		5.37	0.91		1.86	0.34	

M, mean; SD, standard deviation; N = 923; TPI, teacher professional identity; PWB, psychological wellbeing. ***p <0.001; *p <0.05.

**Table 6:** Regional differences in means of teacher professional identity, psychological wellbeing and anxiety.

	***N***	**TPI**		***t***	**PWB**		***t***	**Anxiety**		***t***
		**M**	**SD**		**M**	**SD**		**M**	**SD**	
Urban areas	356	4.19	0.51	0.66	5.47	0.92	1.74	1.81	0.38	−2.13*
Rural areas	567	4.17	0.52		5.37	0.88		1.87	0.38	

*M, mean; SD, standard deviation; N = 923; TPI, teacher professional identity; PWB, psychological wellbeing*.

*p <0.05.

The results revealed that student teachers' professional identity and psychological wellbeing have significant difference (see Table 5). Female individuals' teacher professional identity and psychological wellbeing were significantly higher than male. However, there was no gender difference on anxiety.

The results revealed that anxiety of student teachers from rural areas was significantly higher than urban areas (see Table 6). However, there was no regional difference on teacher professional identity and psychological wellbeing.

## Discussion

As expected, the study revealed the heterogeneity of the current sample in terms of the levels of student teachers' professional identity. By using LPA, this research identified four different profiles (i.e., LPI, MPI, HOV, HPI). The accuracy of the four profiles was supported by the relationships between profile membership (see Table 7). Furthermore, the meaningfulness of the established profiles can be recognized through their relationships with psychological wellbeing and anxiety. Members in LPI profile consistently have a low level of professional identity. Individuals in MPI profile consistently have a moderate level of professional identity. Members in HPI profile consistently have a high level of professional identity. Unlike other three profiles' general disposition, individuals in HOV profile received a higher score in occupational values than other three dimensions.

**Table 7:** Average latent class probabilities for most likely latent class membership (row) by latent profile (column).

	**1**	**2**	**3**	**4**
Profile 1	**0.991**	0.009	0.000	0.000
Profile 2	0.004	**0.992**	0.002	0.002
Profile 3	0.000	0.073	**0.810**	0.117
Profile 4	0.000	0.003	0.042	**0.957**

*N = 923. Values in bold along the diagonal reflect the average probability that participants were correctly categorized in the given latent profile*.

Unlike Karaolis and Philippou's (23) result of three groups (i.e., positive group, negative group, and uncommitted group), the four groups of this results may indicate that Chinese students' teacher professional characteristics may more complex than Western countries. First, the vast majority of Chinese student teachers (94.9%) have a positive evaluation of teaching profession. This may be because the traditional Chinese culture advocates respecting teachers and valuing education (
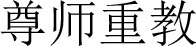
/zun 1 shi 1 zhong 4 jiao 4/), which leads to Chinese teachers' social status is relatively high. Second, other considerations (e.g., financial guarantee of teachers' income with low unemployment). Noteworthy is that, the sharp increase in unemployment caused by COVID-19 may also verify the advantages of being a teacher.

The result revealed that student teachers in HPI profile having a stronger sense of psychological wellbeing and lower anxiety than other profiles, which implicated that student teachers' professional identity is closely related to their mental health. On the one side, the current study found that student teachers' professional identity has a positive correlation with psychological wellbeing, which is consistent with previous studies (37–40). Specifically, student teachers in HPI profile have a higher psychological wellbeing than individuals in other three profiles. On the other side, the finding demonstrates that student teachers' professional identity has a negative correlation with anxiety, which was basically consistent with previous studies (45, 46). In general, student teachers with a high professional identity have a lower anxiety than individuals in other three profiles. This result indicates that teacher professional identity may play a role in protecting student teachers' mental health. One possible explanation may be that student teachers with high psychological wellbeing tend to possess strong self-efficacy beliefs, which may help them to get academic achievement in school (65), and experience enjoyment regarding their future teaching work (66). Another possible explanation may be that teacher professional identity can help individual establish positive self-schemas involving profession-related events successfully, which make individuals more enthusiastic and engage in academic and teacher-training programs actively (51).

The study revealed that female student teachers' professional identity and psychological wellbeing were significantly higher than male, which was in line with previous studies (14, 53). Female may be more encouraged to work as primary and secondary school teachers by society than their male counterparts (49, 53, 67), which may lead them tend to have a higher teacher professional identity. In addition, professional identity may help individuals produce psychological wellbeing (37, 41). Consequently, female student teachers tend to have a higher psychological wellbeing. Consistent with previous research (68–70), this investigation demonstrated that rural student teachers' anxiety was significantly higher than urban student. The reason may be that urban students are more active, alert, and have more opportunities for getting teaching-related skill (70), which may help them react with stressful situations without being anxious. Besides, compared with rural students' parents, urban students' parents are more educated and can help their children deal with stressful situations (68).

## Implications

### Theoretical Implications

First, the results of the current study confirmed four qualitatively different teacher professional identity profiles among student teachers, which indicated that Chinese student teachers may have different characteristic of teacher professional identity compared to Western individuals. The results may also help to demonstrate that the dimensions of teacher professional identity scale (53) are related but also relatively independent. Second, this study explored the unique role of each four dimensions through LPA, correlation analysis, and multiple comparisons. Third, this finding reminded us that different dimensions of teacher professional identity may have different impacts on student teachers' mental health. In particular, it should be pointed out that occupational values and occupational belonging may have different effects under some circumstances that were corroborated.

### Practical Implications

By using LPA, the four sub-group of student teachers' profession identity may offer practical guidelines for teachers and policy makers. First, teachers and policy makers may help student teachers develop teachers' profession identity according different features of sub-group which they belong to. First, 5.1% student teachers belong to LPI, which have a low professional identity with the highest anxiety score. Teachers and policy makers may need to identify these students. Besides, high-quality career guidance even psychological counseling may need to be offered. Second, 54.7% student teachers were grouped into MPI profile and HOV profile, which were characterized by moderate professional identity. For them, cultivating their teacher professional identity may increase the proportion of student teachers engaged in the teaching profession. Third, the results of multiple comparisons have shown that HOV profile's anxiety was not significant with LPI profile. Teachers and policy makers may also need to offer mental health education of student teachers in HOV.

## Limitations and Future Directions

This study has some limitations, which may be addressed through future research. First, the current study only explored the differences in two parameters (i.e., psychological wellbeing and state anxiety) across different latent profiles. However, mental health is a broader concept not only includes psychological wellbeing and anxiety. Future study may consider other predictive constructs of mental health such as subjective wellbeing, trait anxiety or depression. Second, the present study employed a cross-sectional design. Thus, we cannot ascertain causal links between professional identity and wellbeing and anxiety. Future studies should take experimental and longitudinal designs to better explain the causal direction. Finally, this study used self-response measures that can't avoid response bias completely. In addition, the sample we used in this study was entirely Chinese college students, limiting the generalizability of findings across diverse cultures. Future studies should adopt mixed methods to re-test the results.

## Conclusion

This study proved that the sample of student teachers was heterogeneous concerning the levels of their professional identity. By using LPA, we identified four different profiles (LPI, MPI, HOV, and HPI). The LPI, MPI and HPI groups had similar scores in all four dimensions, while HOV group had higher scores in occupational values than other three dimensions. Student teachers' professional identity had positive relationships with psychological wellbeing and negative relationships with anxiety. Psychological wellbeing significantly different across the four profiles.

## Data Availability Statement

The raw data supporting the conclusions of this article will be made available by the authors, without undue reservation.

## Ethics Statement

The studies involving human participants were reviewed and approved by Jiaying University. Written informed consent for participation was not required for this study in accordance with the national legislation and the institutional requirements.

## Author Contributions

SZ: conceptualization, investigation, methodology, validation, formal analysis, writing—original draft, writing—review and editing, data curation, resources, supervision, and project administration. JL: conceptualization, investigation, validation, writing—original draft, writing—review and editing, and resources. YD: writing—original draft, writing—review and editing, software, visualization, and resources. All authors contributed to the article and approved the submitted version.

## Funding

This study was funded by Jiaying University's General Project of Higher Education Teaching Reform (JYJG20190205), Special Moral Education Research Project of the 13th Five-Year Plan for Educational Science of Guangdong Province (2019JKDY014), Educational Science Planning Project of Guangdong (2019GXJK181), Research on the Psychological Mechanism of Hakka Ethnic Group Cognition and Ethnic Identity (21KYK16), and Projects for Humanities and Social Sciences of Jiaying University (2019SKY02).

## Conflict of Interest

The authors declare that the research was conducted in the absence of any commercial or financial relationships that could be construed as a potential conflict of interest.

## Publisher's Note

All claims expressed in this article are solely those of the authors and do not necessarily represent those of their affiliated organizations, or those of the publisher, the editors and the reviewers. Any product that may be evaluated in this article, or claim that may be made by its manufacturer, is not guaranteed or endorsed by the publisher.
